# Prediction of the mobility and persistence of eight antibiotics based on soil characteristics

**DOI:** 10.1016/j.heliyon.2023.e23718

**Published:** 2023-12-15

**Authors:** R.P.J.J. Rietra, B.J.A. Berendsen, Y. Mi-Gegotek, P.F.A.M. Römkens, A.M. Pustjens

**Affiliations:** a Wageningen Environmental Research, Wageningen University & Research, PO Box 47, 6700 AA, Wageningen, the Netherlands; b Wageningen Food Safety Research, Wageningen University & Research, PO Box 230, 6700 AE, Wageningen, the Netherlands

**Keywords:** Antibiotics, Manure, Sorption, Persistence, Fate, Degradation, pH, Soil, Kd, DT50

## Abstract

Antibiotics are widely used in intensive animal husbandry in the Netherlands and are subsequently emitted to soil via manure. To predict degradation and mobility in soil, generic sorption models have been derived. However, most of the coefficients used in generic models are based on a limited range of soils and have not been validated for agricultural soils in the Netherlands. To improve model predictions and assess to what extent differences among soils affect sorption and degradation, an experimental study has been performed. Using a recently developed experimental approach, both the degradation (DT50) and mobility (*K*_d_) of eight selected commonly used antibiotics were determined in 29 typical Dutch agricultural soils. Median DT50 values range from 5.3 days for Sulfadiazine to 120 days for Trimethoprim but are affected by soil type. The ratio of the lowest and highest DT50 for a given antibiotic among soils can be as large as 151, for Tylosin. Measured values of the logK_d_ also range from 0.19 for Sulfadiazine to more than 2 for Doxycycline, Flumequine, Trimethoprim, Tylosin and Enrofloxacine. The impact of soil on *K*_d_ is large, especially for more mobile antibiotics such as Sulfadoxine and Sulfadiazine. Both the range in DT50 and *K*_d_ can be predicted reasonably well using a Freundlich type regression model that accounts for the variation in soil type and sampling depth. Organic matter, iron oxides, pH and clay content appear to be the main constituents and explain between 29 % (Trimethoprim) and 77 % of the variation in DT50 and between 64 % (Lincomycin) and 87 % (Sulfadoxine and Sulfadiazine) of the variation of *K*_d_. The effect of depth on DT50 and *K*_d_ is however limited. The information thus obtained in combination with local data on soil type can be used to more accurately predict the potential risk of relevant antibiotics in soil and transport to ground- and nearby surface waters.

## Highlights

• Variation in degradation (DT50) of eight antibiotics among 29 soils.
• Large variation in sorption (Kd) of eight antibiotics among 29 soils.
• Correlation between Kd and DT50 is low or insignificant for the eight tested antibiotics.
• Soil organic matter, iron oxides, pH and clay are used to predict variety in DT50 and Kd.
• Prediction of the fate of antibiotics in different soils depending on soil characteristics.

## 1 Introduction

Many antibiotics unintendedly enter the soil. They are administrated in animal husbandry and largely excreted through urine and feces. The excreta are directly disposed on soil by grazing animals or applied to soil via fertilization, mainly after manure storage [1]. In the 25 EU/EEA countries sales of antibiotics for food-producing animals fell by 43 % between 2011 and 2020 [2], but selling rates have been projected to increase between 2017 and 2030 by 11.5 % globally [3]. Active substances are usually excreted unchanged; only a small number of antibiotics are metabolized in the animal [4]. After excretion, manure storage [5] and processing (i.e. digestion), can result in (partial) degradation of the active substances [6]. After application of animal manure to the soil, the active substances can either be degraded, adsorbed to soil particles and/or transferred to groundwater and surface water. As a result, both soil and aquatic biota can be exposed to them. To prevent excess exposure, both the exposure route of antibiotics and potential toxic effects on critical biota are modelled in the obligatory preliminary risk assessment (EU Regulation 2019/6 on veterinary medicinal products [7]). At present however, models including those used to predict sorption are rather generic in nature and have not been developed for specific soil types.

Antibiotics can bind to soil particles [8,9], which is reported to decrease in the order of tetracyclines > quinolines > macrolides > chloramphenicol > sulfonamides [10] or fluoroquinolones > tetracyclines > sulfonamide [11]. The binding is influenced by soil type, soil pH, dissolved organic carbon (DOC) and other ions in the soil [9,12]. Most antibiotics tend to adsorb onto the solid soil particles and only sulfonamides tend to leach quickly [11].

In most models developed so far, the soil water partition approach is commonly used. The soil water-partitioning (*K*_d_ in L kg^−1^) is defined by the content of a substance *i* in soil (Q_*i*_ in mg kg^−1^ ds soil), and its equivalent concentration in solution (c_*i*_ in mg L^−1^) with *K*_d_ = Q_i_/c_i_. Since organic matter, or more specific organic carbon therein, is one of the key soil components to which most antibiotics bind, the value of *K*_d_ is often normalized based on organic carbon content with K_oc_ = *K*_d_ f_oc_^−1^, where f_oc_ is the fraction of soil organic carbon. The log K_oc_ of different nonionic organic adsorbents is often correlated with the *n*-octanol/water distribution coefficient (K_ow_) and is compound specific [13,14]. For 66 pharmaceuticals and biocides a close correlation between D_ow_ and K_oc_ (r^2^ = 0.64) was found with log K_oc_ = −0.31 log D_ow_ +2.78, with a standard error of 0.38 on the log K_oc_. This relation holds for monovalent bases with pH_a_ > 5 [15]. To explain the pH effect of the binding of ionizable adsorbates, a pH dependent distribution coefficient was introduced, using the acidity constants of adsorbates [[16], [17], [18]]. For many antibiotics acidity constants are known, showing their ionic character in the pH range from 5 to 7 [19,20] which is relevant for agricultural soils. For example, the sulfonamides registered for animal treatment often contain two acid dissociation constants, which can be assigned to two different sites within the substance (Mangalgiri et al., 2022).

Aside from soil organic matter, antibiotics also can bind to metal(hydro)minerals [9,[21], [22], [23]], as is also demonstrated by the binding to soil before and after removing soil organic matter (SOM) [24]. This suggests that the sorption behavior in soil cannot always be described by the K_OC_ alone and that the potential contribution of soil constituents like clay and oxides should be considered as well. One modelling approach that suitably accounts for the impact of clay and oxides on the binding of antibiotics is the Freundlich sorption model, where sorption to the solid phase can be related to a number of soil properties. In case of the extended Freundlich equation, often referred to as pedotransfer rule, the effect of pH, dissolved organic carbon (DOC), clay, or other soil characteristics, have been used to describe sorption of ionic organic substances to soil [[25], [26], [27]]. Not included in these Freundlich-type models are competition among adsorbates, such as was shown for levofloxacin and phosphate [23] and competition among antibiotics themselves [27]. If proven relevant, this would require the need for alternative sorption models [12].

Even though sorption of reactive chemicals in soil can be strongly non-linear, experimental data for a selection of antibiotics (five sulfonamides, and tetracycline) revealed that Freundlich sorption isotherms are nearly linear with a value of n close to 1 [28], Supporting Information Table A4; [29,30]. For both oxytetracycline and chlortetracycline, however, a value for n of 0.5 was reported [30]. The sorption of sulfonamides is highly influenced by soil pH, with sorption increasing at low pH values [31]. Only for doxycycline n values tend to be larger than 1 [32]. Futhermore, the study by Kodešová et al. (2015) revealed that the Freundlich affinity constant K_F_ of eight pharmaceuticals in thirteen soils were highly affected by soil pH and other soil parameters. This was used to derive pedotransfer models to predict K_F_ using selected soil properties [26]. In addition, pedotransfer models for oxytetracycline and chlortetracycline were developed using SOM, pH and/or Cation Exchange Capacity (CEC) using sorption data from 59 soils [33]. The large differences in selected soil properties for these soils, and the range in effect of pH and SOM further supported the use of Freundlich equation and pedotransfer rules. In addition the Freundlich values close to 1 support the use of the more simple pedotransfer rules to relate *K*_d_ values to soil properties as used previously [34].

Aside from sorption, also degradation is a major process that affects the concentration of antibiotics in soil. In contrast to previous work suggesting that biodegradation was of minor importance for most antibiotics [35], more recent research showed that biodegradation for some antibiotics had to be considered to describe the behavior under field conditions [36,37]. Not surprising though, half-life values (DT50) for antibiotics vary widely (<1–3466 days)(Cycoń et al., 2019), depending on the physical-chemical properties of soil and climatic factors [[36], [37], [38]]. As of now, models to predict DT50 for antibiotics are still scarce and even though predictions of DT50 value exist for substances at screening level value, for example in Estimation Program Interface EPI Suite^tm^ (version 4.11, Washington, DC, USA), it is recommended to use measured half-life values. Ranges in DT50 clearly can be affected by soil composition as well even though the effect can vary. For oxytetracycline and doxycycline for example no or little effect of soil on DT50 was found [39]. On the other hand, additional experimental studies have shown that the half-life values of a number of antibiotics depend on the soil texture. For trimethoprim DT50 ranged from 12 days in a sandy soil to 75 days in a clay soil. Also for tylosin (DT50 ranging from 3 to 73 days in a sand versus clay soil [39], and doxycycline (DT50 ranging from 12 to 21 days in sandy loam and a clay loam respectively; [32]. These reported differences can be due to the binding of the substance to the organic and non-organic soil components, but also due to differences in soil microbiome. As far as we know, no pedotransfers functions have been developed for DT50 data.

Although reliable methods to predict the binding of non-ionizable organic compounds to SOM-on the basis of K_ow_-exist, it is still recommended to experimentally determine the binding experimentally for ionizable compounds. Not in the least since measuring soil adsorption is relatively simple and, as of now, reliable models tested on a range of soils are scarce [15]. To facilitate the initial estimate of both *K*_d_ and DT50 we determined sorption (*K*_d_) and half-live values (DT50) for eight substances in a range of 29 representative soils from the Netherlands used for agriculture. Here, Freundlich type models are derived using multiple linear regression functions with six relevant soil parameters as input variables. We hypothesize that soil characteristics can be used as explanatory variables for antibiotics. The soil characteristics included here are: 1) cation exchange capacity (CEC), 2) clay fraction, 3) pH in soil extract of 0.01 M CaCl_2_ (pH), 4) oxalate extractable iron (Fe_ox_), 5) oxalate extractable aluminium (Al_ox_), and 6) soil organic matter (SOM). To assess whether or not sampling depth affected sorption and or degradation, soil depth of the original soil sample was used as an additional non parametric variable. The nine antibiotics included in the experimental study are: Doxycycline (DOX), Enrofloxacine (ENRO), Flumequine (FLUM), Lincomycin (LINCO), Oxytetracycline (OTC), Sulfadiazine (SDZ), Sulfadoxine (SDX), Trimethoprim (TMP) and Tylosin (TYL) (see Supplementary Information, Table A2 and A3 for characteristics). The substances were also chosen in an earlier study based on the amount used in animal husbandry and/or detection frequency in animal manure (notably SDX, TMP, DOX and OTC [39]; in the Netherlands, and/or detection of the substance in soil and groundwater (in case of LINCO, TYL and SDZ; European Medicines Agency, 2021).

## 2 Material and methods

### 2.1 Reference standards and reagents

The antibiotics OTC, ENRO, FLUM, LINCO, TYL (>85 % TYL A), SDX, and TMP were purchased at Sigma-Aldrich (St. Louis, MO, USA). DOX and SDZ were purchased at Council of Europe (EDQM, Strasbourg, France).

The following internal standards were used: enrofloxacin-d_5_, flumequine-^13^C_3_ and sulfadoxine-d_3_ (Witega, Berlin, Germany), doxycycline-d_3_, lincomycin-d_3_, sulfadiazine-d_4_, tylosin-d_3_, trimethoprim-d_9_ (Toronto Research Chemicals) and oxytetracycline-^13^C_22_^15^N_2_ (RomerLabs Diagnostics, Newark, DE, USA). Methanol (MeOH) was obtained from Actu-All Chemicals (Oss, The Netherlands), ammonia (25 %), citric acid monohydrate, di-sodium hydrogen phosphate and ethylenediaminetetraacetic acid (EDTA) from Merck-Millipore (Burlington, Massachusetts, USA). McIlvain-EDTA buffer: add 500 mL 0.1 M citric acid and 280 mL 0.2 M di-sodium hydrogen phosphate to 1 L Milli-Q water. Adjust the pH to 4.0 using citric acid solution or di-sodium hydrogen phosphate solution and dilute with Milli-Q water to a 2 L volume. Reference standard and internal standard stock solutions were prepared at 1000 mg L^−1^ with the exception of the fluoroquinolones: 100 mg L^−1^. Sulfonamides, trimethoprim and tetracyclines were dissolved in MeOH; (fluoro)quinolones in a solution of 2 % ammonia in MeOH. The macrolide tylosin and the lincosamides were dissolved in Milli-Q water. The mixed native antibiotic solution and the internal standard solution were made at a concentration of 10 mg L^−1^ in MeOH.

### 2.2 Soil samples

Twenty-nine soil samples representing typical Dutch soils were selected from two national soil databases NL1 and NL2 [40]. These databases include soil samples with a wide range in soil properties and represents sandy, clay, peat and loess soils. The land use of the selected soils is largely agriculture including both arable land and grassland. In addition, one urban soil, one forested soil, and a reference soil (OECD) were included. The set contains samples from both the topsoil (0–20 cm) and subsoil (>50 cm, variable depth). The basic characteristics of the selected soils include pH, cation exchange capacity (CEC), soil organic matter (SOM), texture (loam content) and (hydr)oxides (aluminium (hydr)oxide (Al_ox_), iron(hydr)oxide (Fe_ox_). The samples were selected such that a wide range in soil properties was obtained. An overview of the soil parameters, and the sampling locations from all samples is given in table A1 and figure A2 of the supporting information. Soil pH was measured in a 1:10 soil-solution extract of 0.01 M CaCl_2_ [41], CEC was measured after equilibrating the soil three times with 0.1 M BaCl_2_ [42], the clay content (percentage <2 μm) was determined by the pipet method [43], SOM was determined by loss on ignition (LOI) [44,45] metal (hydr)oxides were measured after extraction using a mixture of oxalic acid and ammonium oxalate [46,47]. More details can be found in the source documents describing the samples in the two databases [40].

### 2.3 Degradation study

The degradation study was performed according to a previously published procedure [39]. Eight soil aliquots of 2 g were transferred into a polypropelene tube. Milli-Q water was added to each soil aliquot to achieve a pF of 2. The antibiotic mixture was added to each aliquot to achieve a soil concentration of 100 ng g^−1^ for the individual antibiotics (native mix solution). The fortified soil aliquots were shaken and stored at room temperature under the exclusion of UV light with slightly closed caps to allow airflow into the tubes. Milli-Q water was added weekly to the each aliquot on weight basis to retore the water content. After 0, 1, 2, 3, 7, 10, 21 and 30 days, randomly selected soil aliquots were transferred to −80 °C, assuming this would terminate any potential degradation. After 30 days, internal standards were added and the sample aliquots were extracted and cleaned to proceed with quantitative LC-MS/MS analysis. Degradation kinetics (kinfit) recommended by the Forum for the Co-ordination of pesticide fate models and their Use [48] were applied. For each data points, the remaining fraction of the intact native antibiotic was plotted against the storage time. Four models were tested: single first order (SFO) and three bi-phasic models: first order multi compartment (FOMC), double first order in parallel (DFOP) and hockey stick (HS) (Software: R-studio (Computing, 2017)). If the χ^2^-error of the SFO fit was below 5 %, the SFO model was used. If not, the biphasic model with the lowest χ^2^-error was applied. Based on the selected model, the software automatically calculated the half-life (DT50) of the individual antibiotics. As the timeframe of the experiment is relatively short, for very persistent compounds, severe extraplation occurred yielding high uncertainties for the determined DT50 values.

For some soil samples no reproducible data was obtained, most likely due to an incomplete extraction of insufficient clean-up. As such, in the statistical data analysis only persistence data (DT50) was used that was derived from a model with a maximum χ^2^-error of 10 %. Other data was discarded as the uncertainty in the estimated DT50 was deemed too large.

### 2.4 Mobility study

The mobility of the antibiotics was studied using a column leaching test as was previously reported [39]. Fresh soil (5 g corrected for dry weight) was brought into a reservoir (10 mL, 15 mm diameter, polypropylene) that contained a small layer of cotton wool to keep the soil in place (n = 2). The soil in the reservoirs was wetted by running 5 mL of tap water through it. The individual native antibiotics (200 μL volume containing 400 ng each) were transferred onto the soil. A brief equilibration time to prevent severe degradation of the antibiotics was implemented before 10 mL artificial rainwater (0.01 M CaCl_2_) was run through each soil. The eluate was collected in a centrifuge tube. Next, the soil was removed from the reservoir. Both the water fraction and the soil fraction were analysed by LC-MS/MS. Prior to sample extraction, internal standards were added, to improve the quantification. The *K*_d_ was calculated by dividing the antibiotic content in the soil fraction by the antibiotic content in the water fraction. Also here, in the statistical data analysis only mobility data (*K*_d_) was used that was derived from high quality analytical data. Data were excluded if the positive control (internal standards) yielded low recoveries which prevents sufficiently precise calculation of the *K*_d_. This limitation was mainly observed for peat and heavy clay soils.

As previously discussed (Berendsen et al., 2021), an important limitation of the mobility study (any set-up in which the distribution of substances over multiple phases is determined) is that it is directly influenced by the analytical performance of the methods used to determine the fraction of the substance in the soil and water phase. Especially if the mobility is very high or very low, the concentration of the substance in either fraction might be below the limit of detection. In the case of a nondetect, the limit of detection should be used as the concentration of the antibiotic to calculate *K*_d_. This affects the lower and upper limits of the *K*_d_ value that can result from the chosen approach. To mitigate this limitation, one approach is to apply a very high antibiotic concentration on the soil column. However, this is unrealistic and might influence the *K*_d_. Here, we chose to determine the *K*_d_ at realistic concentrations and acknowledge that any determined *K*_d_ above 2 is to be considered as a rough estimation. Therefore, for antibiotics that strongly bind to almost all tested soils, no meaningful statistical analysis could be carried out.

### 2.5 Sample preparation and analysis

The soil analysis was carried out using a procedure published before for analysis of manure samples [5,49]. In short, 2 g of soil was extracted with 4 mL of a freshly prepared 0.125 % trifluoro acetic acid in acetonitrile and 4 mL of McIlvain-EDTA buffer. In case a larger sample intake was used, extraction volumes were adjusted accordingly. After evaporation of the organic solvent, the extract was cleaned by solid phase extraction (SPE) prior to analysis with liquid chromatography mass spectrometry (LC-MS), either by triple-quadrupole (MS/MS) or by Quadrupole-orbitrap (high resolution MS, hrMS). In the mobility study, the artificial rainwater samples (4 mL) were mixed with 1:1 with McIlvain-EDTA buffer and directly subjected to SPE.

Chromatographic separation was done using a Kinetex C18 2.1 × 100 mm 1.7 μm analytical column (Phenomenex), placed in a column oven operating at 40 °C. The mobile phases used were 2 mM ammonium formate and 0.016 % FA in water (Solvent A) and 2 mM ammonium formate and 0.016 % FA in MeOH (Solvent B). The gradient used at a flow of 0.3 mL min^−1^ was: 0–0.5 min, 1 % mobile phase B, 0.5–2.5 min, linear increase to 25 % B, 2.5–5.4 linear increase to 70 % B, and 5.4–5.5 min linear increase to 100 % with a final hold of 1.0 min. Initial conditions are returned within 0.1 min with a final equilibration time of 0.9 min, resulting in a total run of 7.5 min. The injection volume was 5 μL.

LC-MS/MS analysis was carried out using an Acquity UPLC System, coupled to an AB Sciex Q-trap 6500 mass spectrometer. Both liquid chromatography and mass spectrometry settings, including ion transitions, were used as described previously [5,49]. Data processing was done using MultiQuant 3.0.2 software.

For correct quantification of the soil and water fractions analysed, matrix fortified soil and water were used and isotopically labeled internal standards were added to all individual samples before sample preparation. Analytical methods applied were all ISO 17025 accredited under flexible scope.

### 2.6 Statistical analysis and derivation of relationships bewteen soil properties and Kd or DT50

The relationships to relate *K*_d_ (unit) and DT50 (unit) to soil properties were based on the regression between log transformed values (except for pH) according to equation (1):

[eqn. 1] $$\mathrm{Log}\left[\text{DT}50\right]\text{or}\log\left[\text{Kd}\right]=\mathrm{INT}.+a\cdot \log\left[S_{1}\right]+b\cdot \log\left[S_{2}\right]+\ldots .+e\cdot \log\left[S_{i}\right]$$

Where INT. equals the intercept; a, b, .. e are regression coefficients and S_1_ to S_i_ are soil properties included in the final model.

The final prediction models were generated based on a series of statistical analysis. The independence of the soil properties was assessed by calculating their Pearson bivariate correlation coefficients, and the significance of the correlation coefficients were indicated by their *P*-values. To best describe the relationship between DT50 or Kd and the soil properties, all possible combinations of the soil property variables were considered and different (multiple) linear models to predict DT50 and Kd were generated. The *P*-values were also calculated to assess the significance of the regression models and their explanatory variables. The Akaike's Information Criterion (AIC) score of each generated model was calculated subsequently and all the models were ranked according to their AIC scores. Together with the dependence analysis result of the Pearson correlations and the *P*-values of the regressions, the best models were selected. The effect of soil depth on the mobility and degradation of the selected antibiotics was also evaluated. The soil depth variable, based on the sampling depth, was labeled as shallow or deep and included in the linear regression process. All statistical analyses were performed in R Version 4.1.2.

## 3 Results and discussion

### 3.1 Soil characteristics

Table 1 shows a summary of the selected soil properties used in the experiment. For all soil properties included, the range is such that it covers the normal ranges in Dutch arable and grassland soils [50,51]. Soils range from highly acid (pH < 4) sandy soils with a low organic matter (SOM <1 %) and clay content (clay <4 %) to neutral (pH > 6.5) calcareous clay (clay >25 %) soils as well as peat soils (SOM >20 %).

**Table 1:** Minimum, median and maximum values of soil properties of the 29 selected soil samples included in the study (data on dry weight).

	pH CaCl_2_	CEC^a^ (meq/kg)	SOM** (%)	Clay (% < 2 μm)	Al_ox***_ (mmol/kg)	Fe_ox****_ (mmol/kg)
Minimum	3.1	6.9	0.3	1	1.2	2
Median	5.9	232	10	16	33	88
Maximum	7.8	821	57	53	152	897

a CEC= Cation Exchange Capacity; ** SOM= Soil Organic Matter; *** Alox = aluminium oxides; **** Feox = iron oxides.

In Table 2 the Pearson correlation coefficients between soil properties are shown. Typical correlations are observed such as those between Al_ox_ and clay content, organic matter and CEC suggesting that aluminium in these soils is associated both to the clay minerals and organic matter. Also CEC is correlated strongly to organic matter and clay content, both of which are, in these soils, the dominant soil compounds that contribute to the surface charge properties, more so than oxides of Fe. Soil pH appears not to be correlated strongly to any of the properties included here, which suggests that pH varies independently of clay, SOM and CEC. Only oxides tend to be negatively correlated weakly to pH, which indicates that the presence of oxides is more relevant in acidic soils compared to oxides in pH neutral soils.

**Table 2:** Pearson correlation coefficients between the soil properties.

	Fe_ox_	Al_ox_	Clay	SOM	CEC	pH
Fe_ox_	1					
Al_ox_	0.38***	1				
Clay	0.21**	0.63***	1			
SOM	0.42***	0.64***	0.31***	1		
CEC	0.37***	0.70***	0.61***	0.89***	1	
pH	−0.27***	−0.14*	0.09	−0.17*	−0.03	1

*P < 0.05; **P < 0.01; ***P < 0.005.

### 3.2 Variation in measured DT50 and logKd between soils included in the study

In Table 3 the measured values of DT50 and ^10^logK_d_ are summarized for all soils included. The number of soils included for DT50 ranged from 17 for Doxycycline (DOX) to 25 for Sulfadiazine (SDZ). For Kd between 11 (ENRO) and 27 (FLUM) soils were included.

**Table 3:** Ranges in measured values of DT50 and^10^log transformed values of Kd as well as the number of soils used for each antibiotic and the ratio of the maximum and minimum value.

antibiotic	Class^a^	Median DT50 (days) (min-max; n)	ratio max/min	median ^10^log(Kd) (min-max; n)	ratio max/min
Doxycycline (DOX)	TET	33 (6.9–200; 17)	29	2.07 (1.7–2.5; 16)**	6
Flumequine (FLUM)	QUI	110 (18–380; 23)	22	2.07 (1.0–2.2; 27)**	14
Lincomycine (LINCO)	MAC	47 (7.1–230; 23)	32	0.63 (−0.69–1.6; 27)	186
Oxytetracycline (OTC)	TET	30 (1.9–91; 18)	48	1.71 (0.60–2.2; 20)	35
Sulfadiazine (SDZ)	SUL	5.3 (1.0–23; 25)	23	0.19 (−1.1–1.5; 22)	427
Sulfadoxine (SDX)	SUL	6.6 (1.1–25; 24)	23	0.55 (−1.1–1.9; 22)	1096
Trimethoprim (TMP)	SUL	120 (19–790; 23)	41	2.14 (1.0–2.2; 21)**	17
Tylosine (TYL)	MAC	64 (2.9–440; 23)	151	2.02 (0.28–2.2; 27)	76
Enrofloxacine (ENRO)	QUI	n.a.	–	2.00 (1.5–2.2; 11)^a^	5

a ESVAC classes [2]: Tetracyclines (TET), Quinolines (QUI), Macrolides and lincosamides (MAC), Sulfonamides and trimethoprim (SUL).** In almost all soils these antibiotics strongly bind (*K*_d_ is above 2); no statistical analysis was carried out.

Both DT50 and Kd varied greatly among antibiotics and soils included. The median values of DT50 range from 5.3 days (SDZ) to 120 days (TMP), but also the variation in DT50 for a single antibiotic among soils was large. For most antibiotics the ratio between the maximum and minimum value of DT50 per soil varied between 22 (QUI) to 48 (OTC). For TYL this ratio increased to 151. Note, however, that high values for DT50 are obtained after extrapolation of experimental results, yielding a high measurement uncertainty.

For *K*_d_ the median log transformed value increased from 0.19 for SDZ to values higher than 2 for DOX, FLUM, TMP, TYL and ENRO. The median *K*_d_ increases in the order of SDZ < SDX < LINCO < OTC < ENRO ≈ DOX ≈ FLUM ≈ TMP ≈ TYL, and per class SUL < MAC < TET ≈ QUI ≈ TYL, except that within the class SUL, TMP is not similar to SDZ and SDX. The order in classes is similar to Osterman et al. (2013)(SUL < TET), and Pan and Cu (2017)(SUL < MAC < QUI < TET) except for the order between TET and QUI. As for DT50, the observed variation among soils however was larger and *K*_d_ ranged, on a linear scale from 5 (ENRO) to more than 1000 for SDZ. These experimental data imply that both degradation (DT50) and mobility (*K*_d_) of the antibiotics strongly depend on the soil they are added to. In fact, the measured variation in *K*_d_ among soils was even larger than that of DT50 which implies that sorption of the antibiotics studied here seems to be more variable depending on soil properties than is DT50. For DT50 the observed ranges between minimum and maximum values are, with the exception of TYL, comparable. Also, the variation in DT50 between soils seems not to be related to the median value of DT50. The median of the DT50 is within the range of DT50 values found in literature (Annex A, Table A5 in Suppl Information), except that the DT50 of 120 days for TMP is higher.

In Table 4 the Pearson correlation coefficient (r) is shown between all individual soil properties and the antibiotic's DT50 and *K*_d_, and between DT50 and *K*_d_. For almost all antibiotics, the correlations with individual soil properties are often significant for Kd while the correlations are mostly insignificant for DT50 or have a low significance. The correlation between Kd and DT50 is also insignificant, except for DOX. The significant correlations between DT50 and individual soil properties, and with *K*_d_, are all negative which suggests that with an increase in oxides, clay or organic matter, degradation of the active substances is quicker. This seemingly contradicts with the common notion that half-lives of antibiotics in soils are controlled by availability and sorption [52]. It is well known that DT50 values differ among soils [38] but it is unclear which factors are responsible for this variation. It was previously reported [5] that TYL predominantly degrades by biotic processes. Biotic effects, such as soil respiration or soil microbial biomass, are not represented by any of the soil parameters in this study. Also other well-known factors that influence DT50 values, such as moisture, temperature, concentration, and addition of organic compounds such as manure [36] are not included. The correlation between individual soil properties and *K*_d_ is largely positive except for ENRO. The positive correlation suggests that retention (sorption) increases with an increase in both oxides, clay content and organic matter for eight out of nine antibiotics. Some antibiotics show strong binding in all soils (DOX, ENRO, FLUM, TMP) and therefore, the impact of the range in soil properties is not as pronounced. Furthermore, for ENRO only a limited set of data was available. For *K*_d_, more so than for DT50, the correlation for a single soil property among the antibiotics include here varies more pronounced. For organic matter, for example, the correlation increases from −0.05 for ENRO to +0.75 for SDZ. Also for pH, retention tends to increase with pH except for SDZ and SDX the *K*_d_ of which appears to be negatively correlated to pH. Note that, in contrary to the other antibiotics included, SDZ and SDX have pKa values around 4 and 6 and can be cationic, neutral and anionic in the pH range of the tested soil. Thishis may explain the different behavior of these substances. Increasing the soil pH of two soils from 4.7 to 5.7 to 7.5 and 8.6 respectively, did not change the degradation of fenamiphos, an insecticide with pKa 10.50 [53]. The effect of pig slurry pH on DT50 was compound specific for five compounds [54].

**Table 4:** Pearson correlation coefficient (r) for DT50 and *K*_d_ and selected soil properties.

		Soil properties						
		Fe_ox_	Al_ox_	Clay	SOM	CEC	pH	Kd
DT50	DOX	−0.31	−0.23	−0.37	−0.25	−0.32	−0.44	−0.69*
	FLUM	−0.36	−0.42	−0.51*	−0.18	−0.35	0.09	−0.03
	LINCO	−0.06	0.05	0.19	0.00	0.11	−0.37	0.09
	OTC	−0.38	−0.35	−0.47*	−0.34	−0.42	−0.3	−0.25
	SDZ	−0.26	−0.3	−0.36	−0.31	−0.39	−0.29	−0.23
	SDX	−0.16	−0.19	−0.37	−0.28	−0.39	−0.51*	−0.16
	TMP	−0.41	−0.44*	−0.33	−0.42	−0.47*	0.3	–
	TYL	−0.14	−0.04	0.17	−0.11	0.00	0.19	0.33
	ENRO							–
*K*_d_	LINCO	0.09	0.43*	0.55***	0.24	0.36	0.27	
	OTC	0.23	0.2	0.22	0.12	0.14	0.64***	
	SDZ	0.21	0.64***	0.54	0.75***	0.7***	−0.15	
	SDX	0.22	0.62***	0.54*	0.68***	0.65***	−0.27	
	TYL	0.06	0.29	0.59***	0.06	0.21	0.27	

*P < 0.05; **P < 0.01; ***P < 0.005.

For *K*_d_ three groups can be distinguished based on the correlation; those with a rather strong and positive correlation between *K*_d_ and soil properties (DOX, FLUM, LINCO, SDZ and SDX), those with limited but positive correlation (OTC, TMP and TYL) and ENRO which reveals a largely negative (Except for pH and Fe-ox) correlation between soil properties and *K*_d_. In case of the first group, those with a strong correlation, the relations for SDZ, SDX and LINCO can be explained from their somewhat lower binding affinity (compared to DOX and FLUM). For substances with a lower inherent tendency to adsorb to soil, differences in soil properties such as an increase in soil organic matter, will exert a larger impact on the sorption itself. Substances with a very inherent sorption affinity on the other hand (such as DOX and FLUM) will largely adsorb onto the soil solid phase regardless of the range in soil properties.

The data in Table 4 confirm the previously reported significant interaction between organic matter and antibiotics [24,26]. Therefore, the distribution of organic chemicals between soil and soil solution is often corrected for the amount of organic carbon in soil (K_OC_). The value for *K*_d_ can be recalculated to the corresponding value of K_OC_ via equation (2):

[eqn. 2] $$K_{\text{OC}}=100\;\cdot \;K_{d}/\left(\%\;\text{OC}\right)$$

in Table 5 the ranges in the recalculated values of log K_OC_ are shown as well as the ratio between the maximum and minimum K_OC_

**Table 5:** Overview of calculated values of K_OC_ from *K*_d_.

	DOX	FLUM	LINCO	OTC	SDZ	SDX	TMP	TYL	ENRO
Minimum	2.8	2.6	0.6	2.4	1.1	1.1	2.0	1.8	2.4
Median	3.4	3.3	1.9	3.1	1.6	2.0	3.4	3.1	2.8
Maximum	4.9	4.6	3.9	4.5	2.7	3.2	4.7	4.6	4.0
Max/Min	135	87	2341	132	43	105	498	755	45

**Fig. 1:**
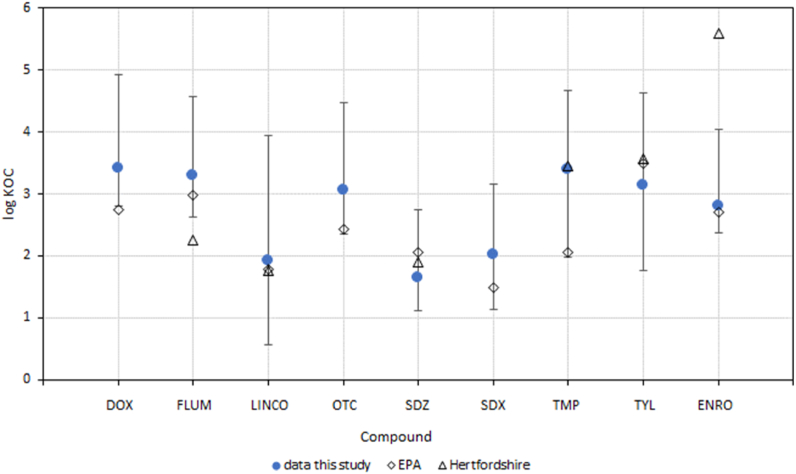
Calculated ranges in log K_OC_ (median: closed symbols and error bars) compared to predicted values by EPA (open diamonds [55]; and those present in the Veterinary Substances Database University of Hertfordshire (open triangles).

The values obtained here, are in agreement with previously published data as shown in Fig. 1. Here the median and range in K_OC_ (min – max) are shown together with predicted values reported in the EPA Comptox database version 2.1 [55,56] and data from the Veterinary Substances Database University of Hertfordshire (http://sitem.herts.ac.uk/aeru/vsdb/index.htm). The prediction for FLUM, SDZ, SDX, and TML inside the reported application domain while the Quantitative Structure-Activity Relationship (QSAR) prediction for DOX, LINCO, OTC, TYL and ENRO are outside the application domain [55]. For 6 out of 9 antibiotics studied here the reported value by EPA is close to the median value measured for Dutch soils. Only for DOX, OTC and TMP, the predicted values from EPA tend to be close to the lower end of the ranges detected in this study.

Also for the data from the Veterinary Substances Database University of Hertfordshire, there is a close match for the value of log K_OC_ for LINCO, SDZ, TMP and TYL and the ones derived from the experimental data from the Dutch soils. For FLUM and, more so for ENRO, the reported log K_OC_ value from the veterinary database is outside the range as determined for the Dutch soils. Especially for ENRO a much stronger retention was reported by the Veterinary Substances Database University of Hertfordshire. Part of the deviation with the experimental data, however, could be due to the extrapolation error resulting from the low concentrations detected.

### 3.3 Impact of soil properties on the variation in DT50 and *K*_d_

Considering the substantial range in measured values of DT50 and *K*_d_, it was hypothesized that part of this variability could be explained by one or more soil properties. Based on [eqn. (1)] we used a multiple linear regression method to describe the variation in the ^10^log transformed values according to:

[eqn. 3] l10og(DT50orKd)=INTERCEPT+a*1+b*log(CEC)+g*log(Feox)+d*log(Alox)+e*log(Clay)+f*log(SOM)+l*pH

In equation (3) the effect of depth is represented by regression coefficient ‘a’. For those chemicals where depth (z) was not significant, the value of ‘a’ is zero. In Table 6 (DT50) and Table 7 (*K*_d_) the best fit models are shown as obtained by multiple linear regression. No constraints have been imposed as to what soil properties are to be included.

**Table 6:** Overview of regression model coefficients and statistical model characteristics (best fit model only) to predict DT50 (non-significant coefficients are marked italic).

	Intercept	a **** depth	b CEC	g Fe_ox_	d Al_ox_	e Clay	f SOM	l pH	R^2^	R^2^adj	se(Y)	F
DOX	3.71***	–	−2.14*	−0.51*	–	*0.47*	1.70*	*0.26*	0.77	0.64	0.22	5.89
FLUM	2.22***	–	–	–	–	−0.29**	–	–	0.30	0.27	0.26	8.28
LINCO	3.11***	–	–	−0.51**	–	0.55**	–	−0.17*	0.42	0.32	0.31	4.11
OTC	2.60***	–	−0.70*	–	–	–	*0*.*37*	–	0.42	0.33	0.34	5.00
SDZ	2.21***	−0.43**	–	–	–	–	−0.21*	−0.18**	0.56	0.49	0.27	8.18
SDX	2.33***	*−0.27*	–	–	–	–	*−0.19*	−0.16*	0.49	0.41	0.27	5.80
TMP	2.25***	–	–	–	–	–	−0.27*	–	0.29	0.25	0.28	7.66
TYL	*0.21*	–	–	–	–	*0.25*	–	0.23**	0.47	0.41	0.33	7.83

*P < 0.05; **P < 0.01; ***P < 0.005.****see equation 3.

**Table 7:** Overview of regression model coefficients and statistical model characteristics (best fit model only) to predict^10^logK_d_ (non-significant coefficients are marked italic). Here regression models for DOX, FLUM and TMP are not shown since most samples had a *K*_d_ of more than 2.

	Intercept	a **** depth	b CEC	g Fe_ox_	d Al_ox_	e Clay	f SOM	l pH	R^2^	R^2^adj	se(Y)	F	p
LINCO	−0.46*	–	–	–	–	1.01***	–	–	0.64	0.62	0.45	40.14	<0.001
OTC	*−0.61*	−0.47*	0.67*		0.71***	−0.86**	–	0.19**	0.85	0.78	0.17	13.32	<0.001
SDZ	*0.38*	–	–	–	–	0.50**	0.50***	−0.18**	0.87	0.85	0.24	36.65	<0.001
SDX	*0.77*	–	*0.30*	–	*0.38*	0.39*	–	−0.33***	0.87	0.83	0.27	24.66	<0.001
TYL^1^	*0.62*	–	−0.57*	*0.28*	–	0.82***	–	0.16*	0.69	0.62	0.32	10.95	<0.001

*P < 0.05; **P < 0.01; ***P < 0.005.****see equation 3.

### 3.4 Predictions of DT50

For two out of eight antibiotics (SDX, SDZ), depth affects the prediction of DT50. This effect of depth is negative, which implies that DT50 for the sulfonamides decreases in the topsoil. This seems logical and would imply that in the topsoil degradation is faster compared to a soil with similar properties in the subsoil. This added effect is possibly related to the microbial activity which is higher in the topsoil compared to the subsoil [57,58].

The effect of organic matter on DT50 is largely negative except for DOX and OTC (not significant). A negative coefficient for organic matter is plausible and is related to the fact that in soils that are rich in organic matter, microbial activity is also higher which could enhance microbial degradation.

The effect of pH is largely negative (3 out of 5 cases), which suggests that degradation occurs faster in high pH soils. It has been reported that an increase in soil pH changes the microbial community, and increase the soil microbial activity [59], which could enhance the degradation of some antibiotics [60]. Also, a decrease in the binding at a higher pH can increase the availability and therefore the degradation. It should be kept in mind that the charge properties of the chemicals included here vary considerably. Where LINCO remains charged positively across the pH range included here, Sulfonamides are neutral to negatively charged. Therefore, the observed impact of pH is in line with the pH dependent charge for Sulfonamides where it is not for LINCO. For DOX, OTC, and TYL, the electrical charge behavior is more complicated and no conclusive single relationship with pH can be expected (see also in the section on prediction of *K*_d_).

This however is in contrast with the predominantly negative effect of Fe_ox_ on DT50. This suggests that in soils rich in iron, degradation occurs faster. However, the increased sorption capacity in soils rich in iron is less relevant. It was observed that the effect of Fe_ox_ on DT50 is present for antibiotics where organic matter has no effect or, in case of DOX, a positive effect (see Table 6). Perhaps this interaction can explain the observation of the negative effect of Fe which is a proxy for that of organic matter.

In contrast to Fe_ox_, Al_ox_ is not significant for any of the chemicals included in the predictions of the DT50. This can be related to the more preferent binding of tetracyclines to divalent iron ions than to trivalent aluminum ions. Also, concentrations of Al_ox_ are lower than those of Fe_ox_ in most soils in this study (Table 1). The regression coefficient for clay is positive for 3 out of 4 chemicals, which indicates that with an increase in the clay content degradation times increase. The CEC effect is however opposite, albeit that the effect of CEC was significant for 2 chemicals only (DOX, OTC). This observation might partly be related to the presence of cationic groups in both tetracyclines, that are cationic until approximately pH 8. The interpretation of the effect of CEC is, however, difficult, firstly because CEC is controlled largely by a combination of clay, organic matter and pH, and secondly because, soil microbial biomass can be related to the soil clay content [61,62]. For DOX and OTC degradation increases with CEC which seems contradictory to the effect of the main constituents of CEC. Possibly the interaction between clay, organic matter, pH and CEC causes this effect.

**Fig. 2:**
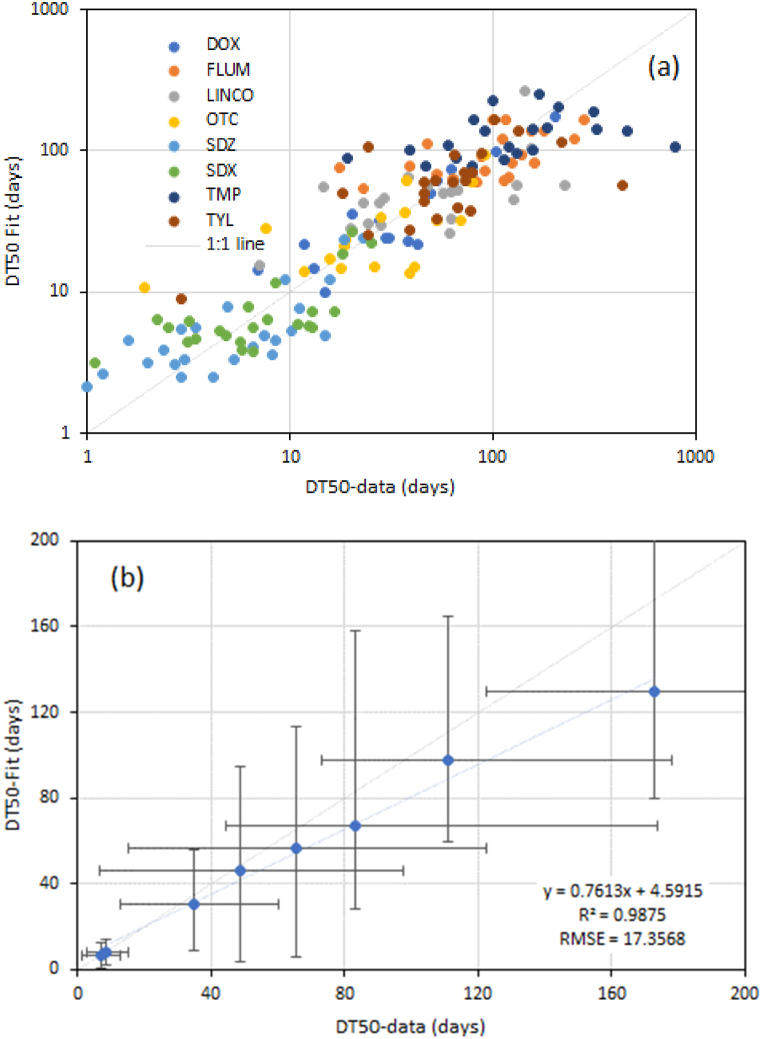
Observed and fitted values (a) and average per antibiotic (b) for DT50 based on the best fit regression models. Error bars represent the standard deviation of measured (X-axis) and predicted (right) DT50.

Based on these results it can be concluded that organic matter, pH and to a lesser extent, Fe_ox_ seem the main parameters that affect DT50 in a more or less consistent way. The effect of depth is moderate, which would suggest that the impact of the higher microbial activity that can be expected in topsoils is not as strong as has been suggested. In general, however, the quality of the regression models is moderate with R^2^ values ranging from 0.29 (TMP) to 0.77 (DOX) as shown in Fig. 2a and b for all chemicals combined.

Data in Fig. 2a and b reveal that the standard error of both measured and fitted values for DT50 are particularly large for TYL, TMP and FLUM, all of which have relatively long degradation times compared to the other five antibiotics included here. Note that the high DT50 empirical values as determined from the degradation experiment are extrapolated. They have a high uncertainty and are more likely to deviate from the regression line.

### 3.5 Predictions of *K*_d_

**Fig. 3:**
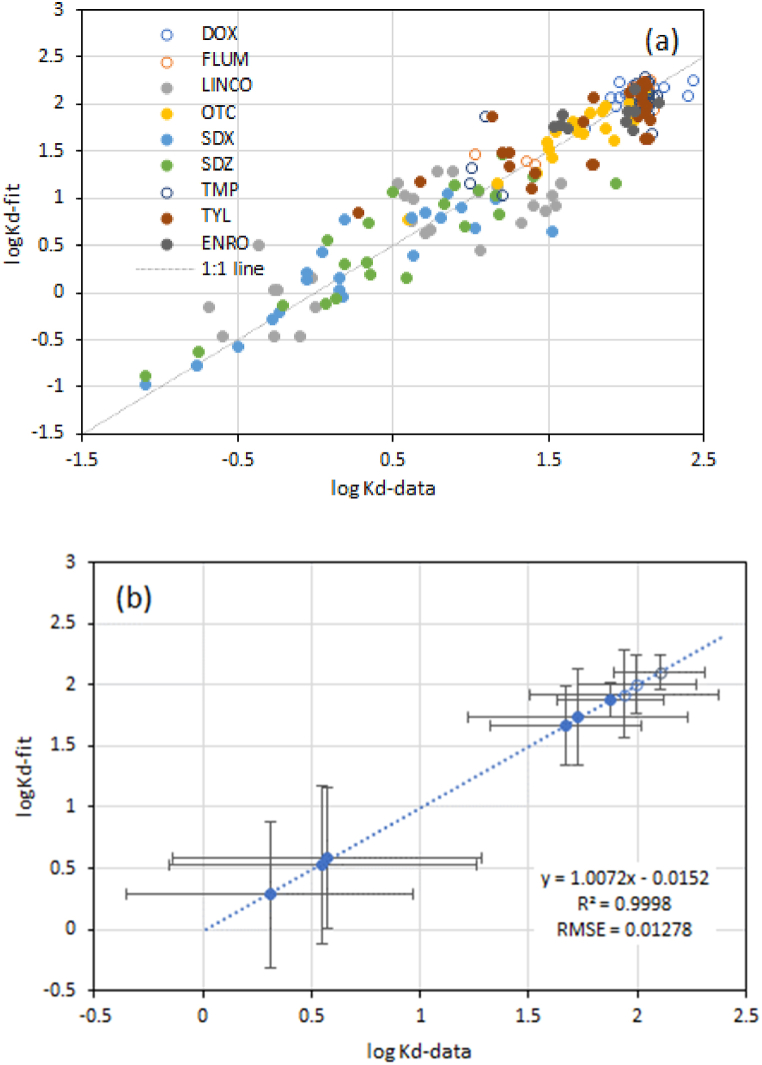
Measured and fitted data (a) and average values (b) of ^10^logK_d_ for the 9 selected antibiotics. Error bars represent the standard error of the measured (X-axis) and fitted (Y-axis) average values. Open symbols refer to antibiotics with an average *K*_d_ of more than 2 (see text).

The quality of the regression analysis to predict *K*_d_ is better than that of DT50 (Fig. 3). Adjusted values of R^2^ for the individual antibiotics range from 0.62 (LINCO) to 0.85 (for SDZ) and p values of the model are all below 0.001. For the prediction of *K*_d_ based on soil properties, depth only has an effect for OTC. The effect of depth is negative, which suggests that sorption decreases with depth for OTC. For *K*_d_, clay content, pH and CEC appear the most relevant soil properties that explain the differences in *K*_d_.

For four antibiotics (LINCO, SDZ, SDX and TYL) the coefficient for clay is positive; for OTC, however, it is negative. This indicates that for most antibiotics *K*_d_ increases with an increase in the soil clay content. OTC is the exception in this study. The effect (sign of coefficient) of CEC is positive except for TYL suggesting that with an increase in the CEC sorption to soil increases. Positive coefficients for SOM in pedotransfer functions of *K*_d_ have been found earlier for antibiotics [26,33,63]. For clay, positive coefficients were found for clindamycin and clarithromycin, positive coefficients were found for pH for clarithromycin [26], and also positive values for coefficients for iron and aluminium (hydr)oxides were found for oxytetracycline and ciprofloxacin [34].

The coefficient for pH is positive for OTC and TYL and negative for the sulfonamides even though it is not always significant. A decrease of the adsorption affinity for sulfonamides at higher pH values is often found in literature [31,64]. With an increase in pH, the net negative surface charge increases and the amphoteric sulfonamides become deprotonated. As such the sulfonamides are repelled by the soil particles and migrate more easily. OTC and TYL can occur as a deprotonated ion and in neutral form within the pH range studied. Tetracyclines, among which OTC, and TYL occur as zwitter ions and therefore their behavior in relation to soil pH is difficult to hypothesize. The data demonstrate that they are more strongly bound at higher pH, indicating that the deprotonated part of the molecule dominates the binding effect.

In contrast to the impact of organic matter to predict DT50, its role is less significant for the prediction of *K*_d_. It is a significant predictor for SDZ only with a positive coefficient which indicates that sorption increases with organic matter. This effect was, however, not observed for the other sulfonamide included here (SDX).

In contrast to the relationships found to explain the variation in DT50, Al_ox_ appears to be a significant property affecting *K*_d_ for two antibiotics with *K*_d_ increasing in soils with a higher concentration of Al_ox_. Whether the observed effects can be related to direct sorption of antibiotics to aluminum(hydr)oxides or that it partially reflects the role of oxides as part of clay minerals and CEC, cannot be deduced from this data. For OTC, which is known to form complexes with divalent ions, the increased sorption with higher mineral content is according to expectations.

Data in Fig. 3a reveal that there is a fair agreement between measured and predicted values of *K*_d_ for all soils. Predictions of the average *K*_d_ across all soils was close to the 1:1 line (Fig. 3b). Samples with measured or predicted *K*_d_ larger than 2 (mostly TMP, DOX and FLUM) are prone to extrapolation errors, and symbols for these 3 compounds are included as open circles to distinguish these from the other antibiotics.

For *K*_d_, the variation in measured and predicted values increases with a decrease in *K*_d_. This suggests that once chemicals tend to adsorb to soils more preferably (log *K*_d_ > 1.50), differences in the sorption capacity as such become less relevant and variation in *K*_d_ between soils decreases accordingly.

## 4 Conclusions

To improve the prediction of the mobility and degradation of nine selected relevant antibiotics in Dutch animal husbandry, sorption and degradation experiments using 29 soils were performed to determine the *K*_d_ and DT50. Results reveal that ranges in DT50 for any given antibiotic across soils are large and observed values for DT50 range with a factor of 23–151 when comparing DT50 for a given compound in the range of soils studied. This ratio exceeds that of the variation in the median DT50 between antibiotics (ranging from 5.3 days to 119 days). This is also true for *K*_d_ where measured ranges in *K*_d_ for a specific antibiotic can range up to a factor 1000 in case of TMP. It should be noted, however, that the error in the measured value of the log *K*_d_ at values higher than 2 can be large due to extrapolation. This is the case especially for DOX, FLUM and TMP. Despite this, the obvious impact of soil properties on both *K*_d_ and DT50 calls for the development of models that are able to account for this. After conversion of *K*_d_ to K_OC_, the recalculated median values of K_OC_ correspond well to previously reported values. Both DT50 and *K*_d_ are partially related to all soil properties included as revealed by Pearson correlation coefficients. Individual correlation coefficients between soil properties and DT50 are largely negative for DT50. In general, DT50 is reduced with an increase in organic matter, pH, iron(hydr)oxides and depth. From these, the impact of organic matter is most likely an indirect one and related to an increase in microbial activity associated with an increase in organic matter. This would explain the seemingly opposite assumed effect of organic matter, where degradation is believed to be reduced with an increase in organic matter.

Most soil properties are positively correlated with *K*_d_, suggesting that retention increases with the amount of the selected properties. This is more in line with the expected interaction of antibiotics in soil where sorption typically increases with organic matter and oxides. Depth was only found to be relevant for 1 antibiotic. The apparent low or high correlation between individual soil properties and *K*_d_ are not consistent with the overall quality of the regression including all soil properties. Interactions between soil properties therefore outweigh the apparent correlation with single soil properties. This is illustrated for OTC which showed rather limited correlation coefficients with individual soil properties (ranging from 0.1 to 0.2), but had a high explained variation when considering all properties in combination (R^2^ 0.85). A comparison of the recalculated values of K_OC_ using the measured soil carbon contact also revealed that the median values for K_OC_ as measured in Dutch soils correspond well for 5 or 6 out of 8 antibiotics with previously published K_OC_ values by EPA and those in the veterinary database collected by the University of Hertfordshire. It should be noted, however, that data on sorption and/or degradation obtained in laboratory experiments, including data from the current study, can deviate substantially from those obtained under field conditions. For example, application of fresh animal manure, which is a common source of antibiotics in soil, delayed the transport of antibiotics [65]. Also long periods with prevailing warm and dry weather conditions caused attenuation of antibiotics in the field [66].

Overall, the generic regression relationships that correct for soil type are able to represent the measured ranges in DT50 and, more so, for *K*_d_ rather well. This suggests that for modeling purposes to predict displacement of antibiotics in Dutch soils improved estimates can be obtained after correction for soil organic matter, clay and oxides and, to a lesser extent pH. As a follow-up of this experiment, the models will be used to simulate the potential transfer of these selected antibiotics to assess whether observed concentrations in soil can be matched by model predictions.

## Funding source disclosure

The research presented in this paper was funded by the investment theme of 10.13039/501100004890Wageningen University and Research (WUR): project KB-34-010-002 and KB-40-003-001.

## Data availability

Data will be made available on request.

## CRediT authorship contribution statement

**R.P.J.J. Rietra:** Writing – review & editing, Writing – original draft, Formal analysis. **B.J.A. Berendsen:** Writing – review & editing, Writing – original draft, Investigation, Funding acquisition, Conceptualization. **Y. Mi-Gegotek:** Writing – review & editing, Formal analysis, Data curation. **P.F.A.M. Römkens:** Writing – review & editing, Writing – original draft, Investigation, Formal analysis, Conceptualization. **A. Pustjens:** Writing – review & editing, Supervision, Project administration, Funding acquisition, Conceptualization.

## Declaration of competing interest

The authors declare that they have no known competing financial interests or personal relationships that could have appeared to influence the work reported in this paper.
