# Supplementary material for: Prediction of the mobility and persistence of eight antibiotics based on soil characteristics
Source: Heliyon. 2023 Dec 15;10(1):e23718. doi: 10.1016/j.heliyon.2023.e23718 (PMC10767508; doi:10.1016/j.heliyon.2023.e23718)
Supplement: Multimedia component 1 [file mmc1.docx]

**Appendix A. Supplementary Information**

Table A1 Soils included in the study including soil classification (68)

| nr | Soil  texture | Soil classification | | Depth  (cm) | pH | CEC (meq/kg) | SOM (%) | clay  (%) | Al_ox_ (mmol/kg) | Fe_ox_ (mmol/kg) |
| --- | --- | --- | --- | --- | --- | --- | --- | --- | --- | --- |
| 2 | clay | eutric fluvisol | | 0-30 | 6.3 | 340.6 | 13.2 | 39 | 67 | 270 |
| 3 | peat | terric histosol | | 0-30 | 6 | 465 | 23.3 | 53 | 141 | 273 |
| 4 | clay | calcaric fluvisol | | 30-90 | 7.6 | 84.6 | 0.5 | 13 | 7 | 16 |
| 5 | loess | haplic luvisol | | 30-100 | 6.7 | 115.9 | 0.6 | 20 | 31 | 33 |
| 7 | peat | terric histosol | | 0-30 | 5.3 | 255.2 | 16.4 | 23 | 152 | 444 |
| 8 | loess | haplic luvisol | | 0-30 | 6.4 | 134.8 | 3.9 | 16 | 32 | 52 |
| 9 | clay | eutric luvisol | | 0-30 | 5.4 | 128.4 | 4.6 | 15 | 33 | 74 |
| 11 | clay | eutric luvisol | | 30-100 | 7.4 | 234 | 3.2 | 30 | 20 | 183 |
| 12 | clay | eutric luvisol | | 0-30 | 6.8 | 367.3 | 10.3 | 32 | 33 | 158 |
| 13 | peat | terric histosol | | 0-30 | 5.6 | 520.9 | 37.6 | 23 | 88 | 188 |
| 16 | peat | terric histosol | | 30-100 | 6.1 | 784.1 | 41.4 | 27 | 150 | 245 |
| 17 | peat | terric histosol | | 0-30 | 4.6 | 229.1 | 20.3 | 9 | 34 | 897 |
| 18 | sand | gleyic podzol | | 30-100 | 7.2 | 36.1 | 1 | 1 | 8 | 9 |
| 19 | sand | gleyic podzol | | 0-30 | 6.9 | 49.1 | 1.8 | 1 | 4 | 9 |
| 20 | sand | terric histosol | | 0-30 | 5.8 | 143.6 | 9.6 | 4 | 19 | 591 |
| 21 | peat | terric histosol | | 30-100 | 5.9 | 820.9 | 57.4 | 8 | 60 | 205 |
| 22 | peat | terric histosol | | 0-30 | 5.1 | 351.4 | 22.7 | 36 | 76 | 298 |
| 24 | sand | aric anthrosol | | 0-30 | 6.3 | 0 | 11.8 | 2 | 0 | 0 |
| 26 | clay | terric histosol | | 0-30 | 5.6 | 415.69 | 13.6 | 39 | 69 | 132 |
| 1 | OECD ref soil | | - | na | 4.8 | 253.88 | 10.4 | 12 | 7 | 2 |
| 29 | clay | terric histosol | | 0-30 | 4.8 | 595.32 | 26.9 | 29 | 53 | 368 |
| 30 | clay | eutric cambisol | | 0-30 | 5.6 | 396.2 | 9.7 | 52 | 59 | 101 |
| 32 | sand | gleyic podzol | | 0-40 | 5.9 | 54.24 | 2.94 | 2 | 16.3 | 38.9 |
| 33 | sand | gleyic podzol | | 75-100 | 5 | 8.34 | 0.33 | 2 | 1.2 | 21 |
| 34 | sand | fimic anthrosol | | 0-40 | 5.4 | 64.71 | 3.37 | 3 | 51.9 | 17.1 |
| 35 | sand | fimic anthrosol | | 60-90 | 4.6 | 6.89 | 0.75 | 3 | 15.8 | 59.5 |
| 36 | sand | haplic arenosol | | 0-10 | 3.1 | 28.38 | 3.54 | 1 | 7.9 | 6.9 |
| 39 | clay | eutric fluvisol | | 80-120 | 7.8 | 294.24 | 2.77 | 28 | 15.6 | 32.8 |
| 41 | loess | haplic luvisol | | 60-90 | 6.7 | 128.48 | 1.83 | 20 | 40 | 43.1 |

Table A2 Structures of used adsorbates.

| Substance (CAS no) | Structure (ECHA ^1^) | Substance (CAS no) | Structure (ECHA ^1^) |
| --- | --- | --- | --- |
| Doxycycline (564-25-0) | 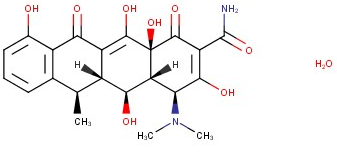 | Sulfadiazine (SDZ) (68-35-9) | 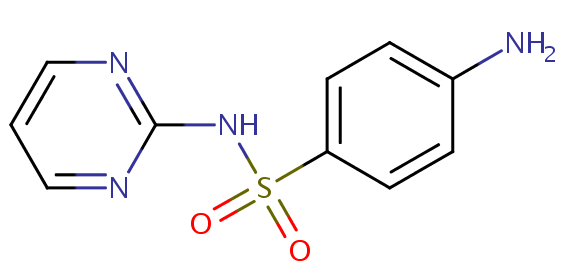 |
| Flumequine (42835-25-6) | 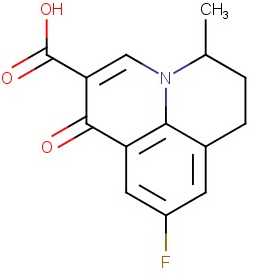 | Sulfadoxine (2447-57-6) | 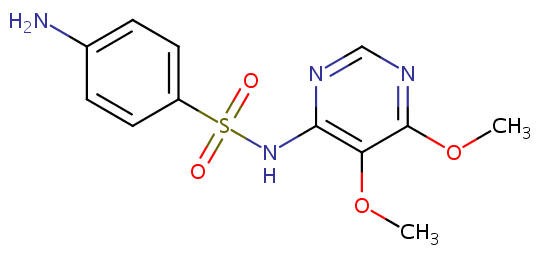 |
| Lincomycin (154-21-2) | 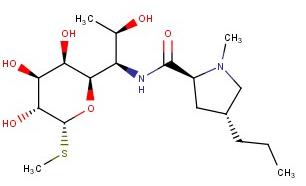 | Trimethoprim (738-70-5) | 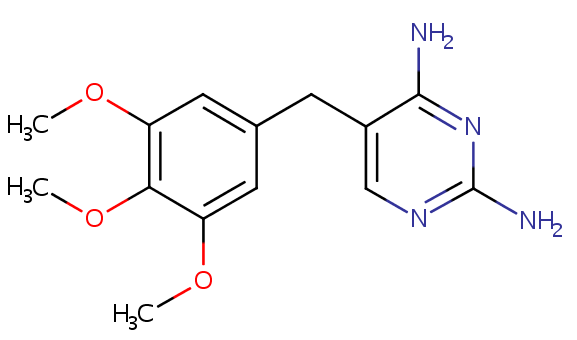 |
| Oxytetracycline (79-57-2) | 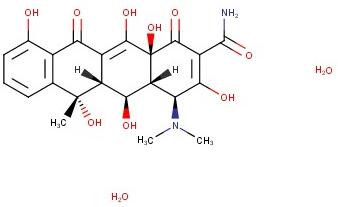 | Tylosin (1401-69-0) | 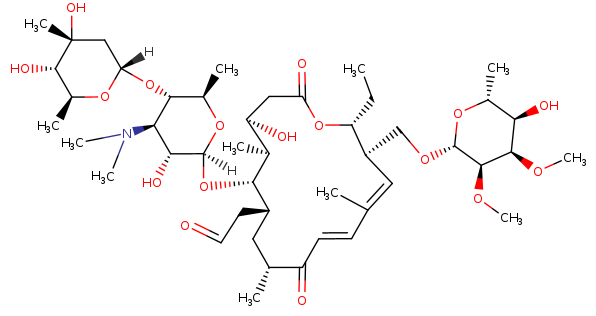 |
| Enrofloxacine (93106-60-6) | 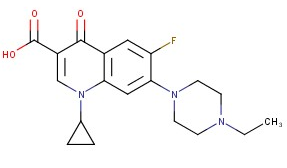 |  |  |

^1^ ECHA (69)

Table A3 Data from used adsorbates (1,2). *Prediction of model outside the reported application domain (57).

| Substance (CAS no) | ESVAC report  Class ^2^ | Molecular  weight | Log K_oa_ | LogK_ow_  Experi-mental | LogK_ow_  Pred-icted | pK_a_ | Ref pK­_a_ | K_oc_ ^4^  (L/kg)  Pred-icted | DT50  Pred-icted^4^ | %  Degra-dation  After  28days  Pred-icted^6^ |
| --- | --- | --- | --- | --- | --- | --- | --- | --- | --- | --- |
| Doxycycline (564-25-0) | Tetracyclines | 444.44 | 9.45 | -0.02 | 0.808 | 2.3  6.49 7.82 | ^3^ | 566* | 147* | 8.1% |
| Flumequine (42835-25-6) | Quinolones | 261.3 | 9.49 | 0.2 | 0.508 | 6.31 | ^6^ | 976 | 7.56* | 6.3% |
| Lincomycin (154-21-2) | Macrolides and lincosamides | 406.5 | 9.50 | 1.6 | 2.08 | 7.79 | ^7^ | 60.3* | 36.8* | 28.3% |
| Oxytetracycline (79-57-2) | Tetracyclines | 460.43 | 9.13 | -0.9 | -0.192 | 2.98  6.87  9.12 | ^3^ | 270* | 148* | 8.1% |
| Sulfadiazine (SDZ) (68-35-9) | Sulfonamides  and trimethoprim | 250.28 | 9.31 | -0.09 | -0.154 | 2.39  6.32 | ^3^ | 115 | 3.36* | 9.3% |
| Sulfadoxine (2447-57-6) | Sulfonamides  and trimethoprim | 310.33 | 9.31 | 0.7 | 0.399 | 2.64  6.43 | ^3^ | 30.8 | 4.29* | 18.5% |
| Trimethoprim (738-70-5) | Sulfonamides  and trimethoprim | 290.32 | 9.34 | 0.91 | 0.887 | 7.10 | ^5^ | 115 | 4.24* | 15.5% |
| Tylosin (1401-69-0) | Macrolides and lincosamides | 916.10 | 9.57 | 1.63 | 2.23 | 7.71 | ^5^ | 3150* | 13.7* | 22.8% |
| Enrofloxacine | Quinolones | 359.39 | 10.4 | - | 0.771 | 5.88  7.74 | ^8^ | 481* | 3.36* | -0.4% |

^1^ (69)

^2^ (2)

^3^ pK_a_ (19), ^5^ (20), ^6^ (70), ^7^ (71), ^8^ (72)

^4^ EPA comptox.epa.gov dashboard (56)

^5^ review of data (37).

^6^ Prediction from (73)

Table A4 Freundlich sorption data of selected eight antibiotics for soil from literature using Q_a_= K_F_ c_a_ ^n^_,_ in which q_a_ is the sorbed amount of substance a (µmol/kg or mg/L), K_F_ (*) and n (-) are the Freundlich parameters, and c_a_ is the concentration in solution after sorption of substance a (µmol/l or ug/g).

| Substance (CAS no) | n | K_F_ | units | Number  of soils | Additional research | reference |
| --- | --- | --- | --- | --- | --- | --- |
| Doxycycline | 1.7-2.5 | 238-308 | gram | 3 | desorption | (33) |
| Flumequine |  |  |  |  |  |  |
| Lincomycin | 0.8-1.0 | 5.09 -210.15 | mg | 3 |  | (74) |
| Oxytetracycline | 0.4 -0.5 | 495-1575 | mg | 2 | Effect of compost and SOM | (64) |
|  | 1.0-1.1 | 1965-3012 | mg | 2 | Effect of pH | (65) |
| Sulfadiazine | 0.9-1.1 | 1.0 - 9.2 | gram | 5 | strong effect of pH | (28) |
| Sulfadoxine | 0.9966 | 0.9311 | gram | 1 |  | (75) |
| Trimethoprim | 0.7 | K_F_ = 9.73 +8.50 C_ox_ ^**^ | mol | 13 |  | (27) |
|  | 1/0.9 – 1/1.08 | 9.46- 10.24 | gram | 3 |  | (26) |
| Tylosin | 0.7 | 2.0-32.1 | gram | 2 |  | (76) |
| Enrofloxacine | 0.4-0.74 | 829-3019 | mol | 20 | Effect of time and desorption | (77) |
|  | 0.265-0.501 | 10^3.634^ – 10^4.069^ | gram | 1 | Effect of pH, Ca, Mg, K, Na, Fe | (78) |

**C_ox_ is soil organic carbon in %

*Unit depends on n: cm^3^/n μg^1-n^ g^-1^ if units are written in gram, L ^1/n^ µmol^1-n^ kg^-1^ if units are written in mol.

Table A5. Data on the biodegradation of selected eight antibiotics for soil (DT50) from literature, based on (37), excluding data on degradation in compost.

| Substance | Half life (days) ^1^ | Half life (days) or other info ^5^ |  |
| --- | --- | --- | --- |
| Doxycycline | 147 | 92% degradation in 49 days | (79) |
|  |  | 533-578 | (80) |
|  |  | 12.28, 13.96 21.01 in sandy loam, loamy soil, and clay soil, respectively | (33) |
|  |  | 9 in sand, and 11 in clay soil | (40) |
| Flumequine | 7.56 | 226 in sand, and 97 in clay soil | (40) |
| Lincomycin | 36.8 | 1.1 in sand, and 11 in clay soil | (40) |
| Oxytetracycline | 148 | 85.6 in compost amended soil  87.3 compost+biochar amended soil | (81) |
|  |  | 35.9-41.3 in manure amended soil  30.2 in non-amended soil | (64) |
|  |  | 21.7 | (82) |
|  |  | 33 in manure-amended soil 56 in non-amended soil | (83) |
|  |  | 86%-95.4% degradation within 120 days | (84) |
|  |  | 8 in sand, and 9 in clay soil | (40) |
| Sulfadiazine | 3.36 | 19 (CaCl2 fraction), 24 (MeOH fraction), 290 (residual fraction), Luvisol  15 (CaCl2 fraction), 13 (MeOH fraction), 490 (residual fraction), Cambisol | (85) |
|  |  | 8.48 to 10.22 non-sterile soil  30 to 21.21 sterile soil | (86) |
|  |  | <1 and 8.5 for low and high addition in loamy sand  <1 and 5.6 for low and high addition in silt loam | (87) |
|  |  | Recovery 27-45% after 1 day  Recovery 15-18% after 32 days, and 7-10% for high and low treatment, respectively | (88) |
|  |  | Degradation rate constant (day) 0.115 and 0.041 at low and high addition | (89) |
|  |  | <0.002 mg/kg 63 days | (90) |
|  |  | 4.8 | (91) |
|  |  | 0.6 in sand, and 1.0 in clay soil | (40) |
| Sulfadoxine | 4.29 | 0.9 in sand, and 1.4 in clay soil | (40) |
| Trimethoprim | 4.24 | 26 and 26.1 for aerobic and anaerobic soil, resp. | (92) |
|  |  | 2-5 | (93) |
|  |  | Degradation 13-84% after 61 days | (39) |
|  |  | 12 in sand, and 75 in clay soil | (40) |
| Tylosin | 13.7 | 7-8 | (94) |
|  |  | 8 | (95) |
|  |  | Degradation 100, 100 and 60% after 30 days at 30, 20, and 4°C | (96) |
|  |  | Degradation 100% after 13 days | (97) |
|  |  | 2 in field without history and 10.2 in field with history of exposure | (98) |
|  |  | 4.4 in laboratory, and 6.1 in field | (99) |
|  |  | 67 and 49 and low and high addition | (100) |
|  |  | 8 | (93) |
|  |  | Degradation 100% after 13 days | (101) |
|  |  | Degradation 50% after 4.2 days in sand and 5.7 days in sandy loam | (76) |
|  |  | 3 in sand, and 73 in clay soil | (40) |

^1^ EPA: <https://www.epa.gov/> dashboard chemical env-fate-transport (56) . From OPERA Calculation Report.


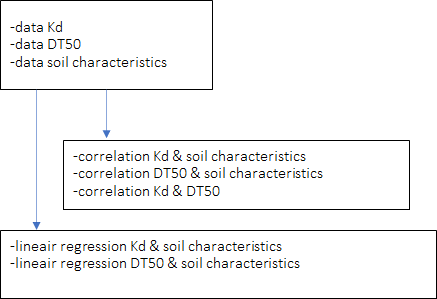


Figure A1 Methodology flow sheet


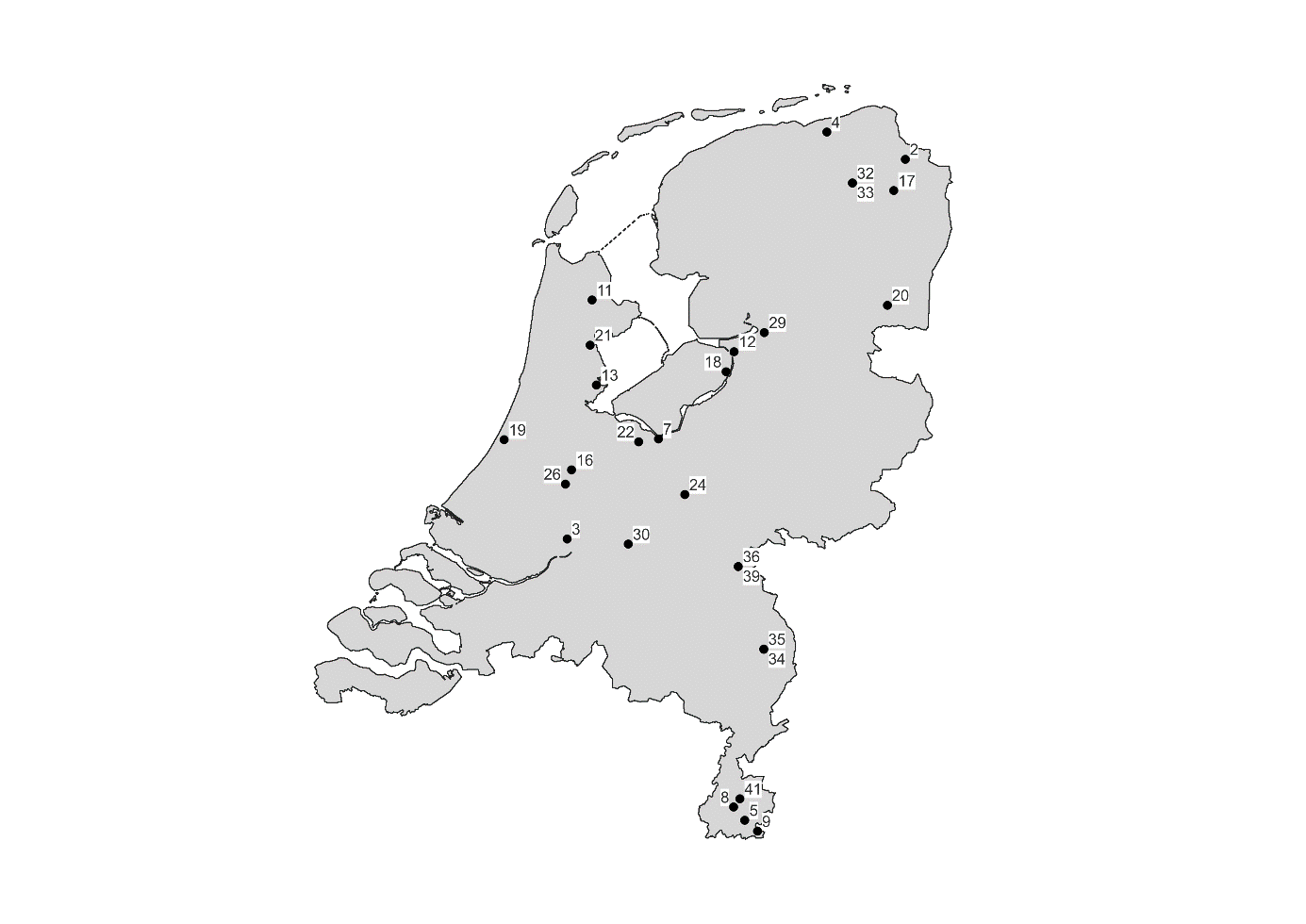


Figure A2 Sampling locations including number from Table A1
